# MRTX1133 attenuates KRAS^G12D^ mutated-colorectal cancer progression through activating ferroptosis activity via METTL14/LINC02159/FOXC2 axis

**DOI:** 10.1016/j.tranon.2024.102235

**Published:** 2024-12-09

**Authors:** Junwei Zou, Xiuhua Shi, Zhaoying Wu, Siyuan Zuo, Xiaolei Tang, Hailang Zhou, Yong Huang

**Affiliations:** a Department of Gastrointestinal Surgery, The Second Affiliated Hospital of Wannan Medical College, Wuhu, Anhui, China; b Department of Radiotherapy & Oncology, The No.2 People's Hospital of Wuhu City, Wuhu, Anhui, China; c School of Clinical Medicine, Wannan Medical College, Wuhu, Anhui, China; d Center for Translational Medicine, The Second Affiliated Hospital of Wannan Medical College, Wuhu, Anhui, China; e Department of Gastroenterology, Lianshui People's Hospital of kangda college Affiliated to Nanjing Medical University, Huai'an, Jiangsu, China

**Keywords:** Colorectal Cancer, KRAS^G12D^, MRTX1133, ferroptosis, LINC02159, METTL14, FOXC2

## Abstract

Colorectal cancer (CRC) ranks as the third most commonly diagnosed cancer and the second leading cause of cancer-related deaths worldwide. Studies have shown that CRC patients with KRAS mutations, especially KRAS^G12D^, have an increased risk of metastasis. Emerging evidence indicates that long non-coding RNAs (lncRNAs) are crucial in the carcinogenesis and progression of various cancers, regulating multiple biological processes but the link between KRAS^G12D^ mutations and lncRNAs in CRC remains unclear. Therefore, this study was designed to identify a novel lncRNA involved in KRAS^G12D^-mutated CRC and to elucidate its molecular mechanisms. The analysis of differentially expressed lncRNAs in the GSE201412 dataset revealed that LINC02159 was significantly upregulated following treatment with the KRAS^G12D^ inhibitor MTRX1133 Data from the GTEx database indicated that LINC02159 is highly expressed in CRC tumour tissues and is associated with better patient outcomes. In vitro and in vivo experiments suggest that LINC02159 acts as a tumour suppressor in CRC progression. Specifically, LINC02159 knockdown negated the inhibitory effects of MRTX1133 on tumourigenesis and its promotive effect on ferroptosis in KRAS^G12D^-mutated CRC cells. LINC02159 expression is regulated by METTL14, with METTL14 knockdown decreasing m6A methylation of LINC02159, leading to its increased expression in CRC cells. Additionally, LINC02159 stabilised FOXC2 expression through de-ubiquitination. Rescue experiments further clarified that the METTL14/LINC02159/FOXC2 signalling axis is crucial for the inhibitory effects of MRTX1133 in KRAS^G12D^-mutated CRC. Our study provides novel insights into the therapeutic potential of MRTX1133 in treating KRAS^G12D^-mutated CRC by identifying a METTL14/LINC02159/FOXC2 signalling axis that mediates drug response. Our findings highlight the importance of understanding the molecular mechanisms of lncRNAs in cancer to develop effective targeted therapies.

## Introduction

In 2020, colorectal cancer (CRC) ranked as the third most frequently diagnosed cancer and the second leading cause of cancer-related deaths globally, with nearly 2 million new cases and 1 million fatalities[1]. Currently, radical surgery remains the most prevalent treatment for CRC. However, most CRC patients are diagnosed at advanced stages, rendering them poor surgical results and even loss of surgical opportunities, so chemotherapy and other treatment modalities are commonly utilised to enhance clinical outcomes [1].

CRC is a highly complex and multistep process driven by a combination of genetic mutations and epigenetic alterations. Its molecular pathogenesis is marked by the disruption of several cellular pathways including cell proliferation, apoptosis, differentiation, and DNA repair mechanisms. CRC progression is primarily influenced by two critical factors: genetic mutations and the instability of epigenetic regulation [2,3]. Numerous studies have shown that CRC patients harbouring KRAS mutations have an increased risk of metastasis [[4], [5], [6], [7]]. Approximately 40% of CRC patients exhibit KRAS mutations, including variants such as KRAS^G12D^, KRAS^G12V^, and KRAS^G13D^, with the KRAS^G13D^ mutation, in particular, associated with poorer prognoses, including a higher likelihood of metastasis and reduced survival time [[8], [9], [10]]. The KRAS^G12D^ mutation is associated with an increased risk of metastasis and poorer prognosis [11]. Furthermore, the immune environment of KRAS^G12D^-mutated metastatic CRC is characterised as ‘immune excluded’ [12,13] indicating that immunotherapy may be particularly challenging for this cancer subtype. Also, the therapeutic efficacy of cetuximab in patients with KRAS mutations remains limited [14], therefore, there is an urgent need to investigate new strategies for targeting metastatic CRC in patients with KRAS^G12D^ mutations.

Long non-coding RNAs (lncRNAs) are a diverse group of transcripts exceeding 200 nucleotides in length, possessing minimal or no protein-coding potential [15]. Increasing evidence indicates that lncRNAs are crucial in the carcinogenesis and progression of various human cancers, as they regulate multiple biological processes including metabolism, invasion, and metastasis [16]. However, the correlation between KRAS^G12D^ mutations and lncRNAs in CRC remains unclear. Therefore, this study was designed to identify a novel lncRNA involved in KRAS^G12D^-mutated CRC and elucidate the underlying molecular mechanisms. The analysis of differentially expressed lncRNAs in the GEO dataset GSE201412 revealed that LINC02159 was significantly upregulated following treatment with the KRAS^G12D^ inhibitor MTRX1133. Data from the GTEx database indicated that LINC02159 was highly expressed in CRC tumour tissues and correlated with better patient outcomes. In vitro and in vivo experiments suggested that LINC02159 acts as a tumour suppressor in CRC progression. Specifically, LINC02159 knockdown negated the inhibitory effects of MRTX1133 on tumourigenesis and its promotive effects on ferroptosis in KRAS^G12D^-mutated CRC cells. LINC02159 expression was modulated by METTL14 and METTL14 knockdown decreased m6A methylation of LINC02159, leading to increased LINC02159 expression in CRC cells. Additionally, LINC02159 stabilised FOXC2 expression through de-ubiquitination. Rescue experiments further clarified that the METTL14/LINC02159/FOXC2 signalling axis is crucial for the inhibitory effects of MRTX1133 in KRAS^G12D^-mutated CRC. Our findings provide novel insights into the therapeutic potential of MRTX1133 in treating KRAS^G12D^-mutated CRC.

## Materials and methods

### Clinical samples

This study included 18 patients who underwent radical resection at the Second Affiliated Hospital of Wannan Medical College between 2022–2023. None of the patients received chemotherapy or radiotherapy before surgery. Tumour tissues were promptly snap-frozen in liquid nitrogen post-resection and preserved at -80 °C until further use or paraffin embedding. The research protocol was approved by the Ethics Committee of the Second Affiliated Hospital of Wannan Medical College (WYEFYLS2024137), and informed consent was obtained from all participants.

### Cell culture and treatment

The cell lines HT29, DLD1, RKO, HCT116, SW480, Caco-2, LS174T and LS180 were acquired from the Chinese Academy of Sciences (Shanghai, China). LS174T cells were cultured in DMEM supplemented with 10% fetal bovine serum (FBS; Thermo Fisher Scientific, Waltham, MA, USA) and 1% antibiotic mixture containing 100 U/mL penicillin and 100 mg/mL streptomycin (P/S) (Beyotime, C0222). HT29, DLD1, SW480, and HCT116 cells were cultured in RPMI-1640 medium (HyClone, SH30809.01) supplemented with 10% FBS and 1% P/S. Caco-2 cells were maintained in Ham's F-12K medium (Gibco, 21127022) with 10% FBS and 1% P/S. RKO, LS174T and LS180 cells were maintained in MEM (Gibco, 11095080) containing 20% FBS and 1% P/S. KRAS^G12D^-mutated CRC cells were treated with MRTX1133 as previously described[17].

All drugs were obtained from MedChemExpress unless otherwise specified and they were used according to the manufacturer's instructions. Small interfering RNAs (siRNAs) were sourced from TsingKe Technology (Beijing, China). Transfection of siRNA (50 nM) into colorectal cells was performed using Lipofectamine 2000 (Thermo Fisher Scientific), with nonspecific siRNA (50 nM) serving as the negative control. The siRNA sequences are as follows:

si-control (CTL):5′-UUCUCCGAACGUGUCACGUTTACGUGACACGUUCGGAGAATT-3′;
si-LINC02159#1: 5′-CAGCCCUGCACAUUAUGUATTUACAUAAUGUGCAGGGCUGTT-3′; si-LINC02159#2: 5′-CGCACUUAGAGAGAGUAAATTUUUACUCUCUCUAAGUGCGTT-3′; si-WATP: 5′-GGUUCGAUUGAGUGAAACAUU-3′;
si-FTO: 5′-UGGAUUACCAAUGAGGAUGCG-3′;
si-METTL3: 5′-CTGCAAGTATGTTCACTATGA-3′;
si-METTL14: 5′-GCAGCACCUCGGUCAUUUA-3′;
si-ALKBH5: 5′-GCUUCAGCUCUGAGAACUATT-3′.

### CRISPR-Cas9 functional genetic screen

LS174T and LS180 cells were transfected with a lentivirus expressing a CRISPR library targeting the human kinome at a multiplicity of infection (MOI) of 0.5 ensuring 500-fold coverage. Following a seven-day selection period with puromycin (2 µg/mL), the surviving cells were harvested and genomic DNA (gDNA) was extracted (T0) from a portion equivalent to 500-fold coverage of the library. The remaining cells were cultured in 15 cm dishes either with or without 25nM MRTX1133. The medium was refreshed every three days and cells were passaged upon reaching confluence. After ten days of drug treatment, cells were harvested, and gDNA was extracted using the Blood and Cell Culture DNA Maxi Kit (69,506, Qiagen) according to the manufacturer's instructions. The kinome CRISPR library was then prepared from the extracted gDNA and subjected to HiSeq analysis. The CRISPR viability score was calculated as the mean of the log2-transformed fold change of all single guide RNAs (sgRNAs).

### Animal models

To investigate the effects of LINC02159 knockdown on tumour growth, LS180 cells were infected with lentiviral vectors (GV248, GeneChem, Shanghai, China) expressing either LINC02159-targeting shRNA or a scrambled control sequence. The infection was performed at MOI=20 with 5 μg/mL polybrene in antibiotic-free DMEM supplemented with 10% FBS. After 24 h, the medium was replaced with fresh complete medium, and infected cells were selected with 2 μg/mL puromycin (Sigma-Aldrich, P8833) for 2 weeks. For xenograft experiments, these stable cells were harvested at 80% confluence using trypsin/EDTA solution (Gibco, 25,300,054), then resuspended in PBS to a final concentration of 1 × 10^[8] cells/mL and a 100 μL of cell suspension was subcutaneously injected into the right flank of approximately 6-week-old female BALB/c nude mice (n = 4 per group; Vital River Laboratories, Beijing, China). All mice were sacrificed 28 days post-inoculation and the tumours were harvested, measured and weighed. The mice were housed under specific pathogen-free conditions and all animal experiments were approved by the Second Affiliated Hospital of Wannan Medical College Animal Care and Use.

### Quantitative real-time polymerase chain reaction (qRT-PCR)

Total RNA was isolated using the TRIzol reagent (Invitrogen, USA) and reverse transcribed. RT-qPCR was performed using the SYBR Green Master Mixture (Vazyme, China), with GAPDH serving as the internal control. The relative gene expression levels were quantified using the 2^−ΔΔCt^ method and each assay was conducted in triplicate. The primer pairs were as follows:

LINC02159,
Forward;5′-CCACCCCTTTCCCTGTAAGAG-3′,
Reverse; 5′- TTGGTCAAAGCCAAAAGCCG-3′,
U6,
Forward; 5′-CTCGCTTCGGCAGCACA-3′,
Reverse; 5′-AACGCTTCACGAATTTGCGT-3′,
GAPDH,
Forward; 5′-CTCTGATTTGGTCGTATTGGGC-3′,
Reverse; 5′-CCTGGAAGATGGTGATGGGATT-3′.

### Western blotting

Cell extracts were prepared using RIPA lysis buffer (Beyotime, China) and the protein was quantified with a BCA Protein Assay Kit (Beyotime, China). Proteins (40 μg) were separated on 12% SDS-polyacrylamide gels and transferred to PVDF membranes (Bio-Rad, Hercules, CA). The membranes were blocked with 5% fat-free milk for 1 h at room temperature, followed by overnight incubation with primary antibodies at 4 °C (GAPDH CST, 2118S, 1:1000; anti-METTL14 CST, 51104S, 1:1000; anti-FOXC2 CST, 8186S, 1:1000). The membranes were then incubated with HRP-conjugated secondary antibodies for 1 h at room temperature and the protein bands were visualised using an ECL kit (Merck Millipore, USA).

### CCK-8 assay

Cell proliferation was assessed using the Cell Counting Kit 8 (MCE, New Jersey, USA) to determine cell viability. The spectrophotometric absorbance of each sample at 450 nm was measured using the Infinite M200 spectrophotometer (Tecan).

### EdU assay

The EdU cell proliferation assay was conducted using the BeyoClick™ EdU Cell Proliferation Kit with Alexa Fluor 555 (Beyotime) following the manufacturer's instructions. Briefly, cells were incubated with 10 μM EdU working solution at 37 °C for 2 h, then fixed using a fixative solution and washed three times. Subsequently, the cells were treated with click additive solution and stained with Hoechst 33,342. The stained cells were then observed using an LSM 5 Pascal Laser Scanning Microscope (Carl Zeiss, Oberkochen, Germany).

### Transwell assay

For the migration assay, transfected CRC cells were transferred to the upper chamber without Matrigel (Corning, NY, USA) and cultured in 0.1% DMEM medium (Gibco, 11,965,092) containing 0.1% FBS. In invasion assays, cells were seeded into the upper chambers pre-coated with Matrigel (Corning, NY, USA), while the bottom chamber was filled with 10% DMEM medium. After 36 h, the chambers were fixed with 4% paraformaldehyde (Beyotime, China) and stained with 0.1% crystal violet (Solarbio, China) for observation using a Carl Zeiss inverted microscope.

### Subcellular fractionation

Cytoplasmic and nuclear RNA were isolated using the Cytoplasmic & Nuclear RNA Purification Kit (Norgen Biotek, Canada) according to the manufacturer's instructions. The isolated RNA was then reverse transcribed for qRT-PCR. The cytoplasmic and nuclear proteins were separated using the Nuclear and Cytoplasmic Extraction Kit (Cwbio, China).

### RIP

The RIP assay was conducted using a RIP kit (Genseed, China). Approximately 1 × 10^[7] cells were harvested and lysed in RIP lysis buffer. Magnetic beads were incubated with 5 µg of anti-FOXC2 or control IgG at 4 °C for 2 hours. The cell lysates were then incubated with the antibody-bound magnetic beads at 4 °C overnight. The immunoprecipitated RNA complexes were subsequently purified and quantified by qRT-PCR.

### Methylated RNA immunoprecipitation assay (MeRIP)

An anti-N6-methyladenosine (m6A) antibody (ab151230) was used to pull down m6A-modified LINC02159. Total RNA was extracted from cells using Trizol (Thermo Fisher) and 100 μg of RNA was added to 500 μl of MeRIP buffer (150 mM NaCl, 10 mM Tris–HCl, pH 7.5, 0.1% NP-40) and briefly incubated with 1 μl of rabbit IgG (Jackson ImmunoResearch, 111–035–003). The IgG was then removed using protein A/G beads (Santa Cruz, sc-2003). The pre-cleaned lysates were transferred to new tubes and incubated with rabbit IgG or m6A antibody for 2 h at 4 °C with rotation. Finally, the m6A-bound RNA was extracted with Trizol and the RNA level of LINC02159 was measured by qRT-PCR.

### Ferroptosis activity

Treated cells were trypsinised and resuspended in a medium containing 10% FBS before the addition of 10 µM of C11-BODIPY (ThermoFisher Scientific, California, USA) for 30 min at 37 °C in a 5% CO_2_ atmosphere shielded from light. Post-incubation, the cells were washed twice with PBS to remove excess C11-BODIPY and the fluorescence was assessed by concurrently acquiring green (484/510 nm) and red (581/610 nm) signals using a flow cytometer.

The Iron Assay Kit (Sigma Aldrich, Shanghai, China) was used to quantify Fe^2+^ or total Fe in various cell lines. Initially, 2 × 10^[6] cells were rapidly homogenised in 4–10 vol of Iron Assay buffer and centrifuged at 13,000 × g for 10 minutes at 4 °C to remove insoluble materials. For ferrous iron (Fe^2+^) measurement, 5 µl of iron assay buffer was added to each well. For total iron measurement, two sets of wells were prepared: one set received 5 µl of assay buffer, while the other received 5 µl of Iron Reducer to convert Fe^3+^ to Fe^2+^. Subsequently, 5 µl of Iron Reducer was added to all sample wells to reduce Fe^3+^ to Fe^2+^. The samples were thoroughly mixed using a horizontal shaker or pipetting and incubated in the dark at room temperature for 30 min before the addition of 100 µl of Iron Probe. The samples were mixed well using a horizontal shaker or pipetting and incubated for 60 min at room temperature in the dark and the absorbance was measured at 593 nm (A593).

The GSH test kit (Jincheng, Nanjing, China) was employed to measure GSH/GSSG concentrations in cells. The necrosis and apoptosis detection kit (C1056, Beyotime, China) was used to assess cell viability to evaluate the survival status of cancer cell lines under ferroptosis stress induced by Erastin or RSL3 in vitro. All experiments were conducted in triplicate (n=3).

### TRAP and LC-MS/MS

A TRAP Kit (Bersinbio, China) was utilised to investigate the interaction between lncRNA and proteins. Control and LINC02159 overexpression vectors containing MS2 stem-loop structures (MS2 and lncRNA-MS2) and a GST-MS2 overexpression vector were designed by Bersinbio. MS2 and lncRNA-MS2 vectors were co-transfected with GST-MS2 into LS180 cells to form the GST-MS2∼lncRNA-MS2 complex and interacting proteins. After transfection, the cells were lysed, and the complex was extracted using glutathione affinity magnetic beads to isolate the target proteins. The lncRNA-binding proteins were identified by mass spectrometry and the TRAP experiment was validated by western blotting. Protein samples digested with sequencing-grade trypsin were analysed by LC-MS/MS to obtain the original mass spectrometry data. The raw files were analysed with Byonic software to identify the proteins in the UniProt-Homo sapiens database.

### FISH

Cells cultured on slides were washed with PBS and then fixed with 4% paraformaldehyde. Following treatment with a protease reagent, the slides were exposed to a prehybridization buffer at 40 °C for 4 h, followed by an overnight hybridization with a digoxin-labeled probe at the same temperature. Post-washing and blocking, biotin-conjugated anti-digoxin antibody was applied. Subsequently, the slides were incubated with CY3 at 37 °C for 30 min after washing. Image capture was performed using an Olympus inverted fluorescence microscope. The probe, sourced from Boster Bio-tech in California, USA, was used to evaluate all fields.

### Statistical analysis

Statistical analyses were conducted using GraphPad Prism 9.0 and R software. Comparisons between two groups were performed using Student's *t*-test. A p-value of less than 0.05 was considered statistically significant. Data was presented as the means± SD of at least three independent experiments.

## Results

### LINC02159 is upregulated in KRAS^G12D^ mutated CRC cells by MRTX1133 treatment

**Fig. 1:**
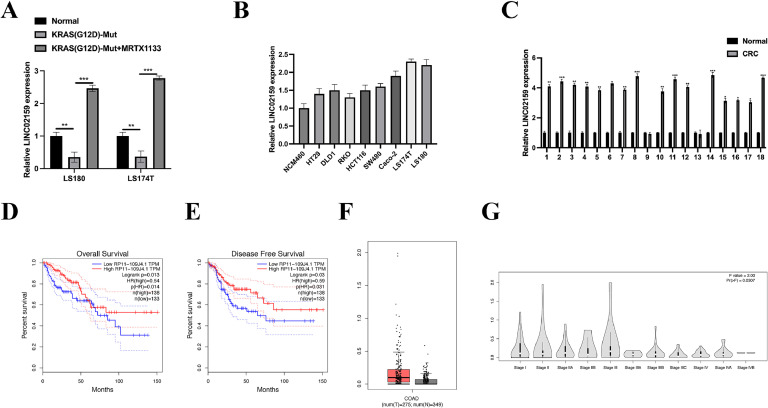
**LINC02159 is upregulated in MRTX1133-treated CRC cells.** A: qRT-PCR was performed to evaluate LINC02159 expression in both LS180/LS180-KRAS^G12D^ and LS174T/LS174T-KRAS^G12D^ cell lines following MRTX1133 treatment. B: LINC02159 expression in CRC cell lines and NCM460 cells was quantified by qRT-PCR. C: LINC02159 expression in 18 pairs of CRC tumour tissues and adjacent normal tissues was detected by qRT-PCR. Overall survival rate (D), disease-free survival rate (E), LINC02159 expression in CRC tissues and normal adjacent tissues in GEPIA (F), and LINC02159 expression in CRC tissues from different stages (G) from the GTEx database. **P<0.01, ***P<0.001. Data are presented as mean ± SD.

The analysis of the public dataset GSE201412^17^ revealed a marked upregulation of LINC02159 in CRC cells harbouring the KRAS^G12D^ mutation compared to the control group. This finding was corroborated through experimental validation in both LS180/LS180-KRAS^G12D^ and LS174T/LS174T-KRAS^G12D^ cell lines following MRTX1133 treatment (Fig. 1A). Furthermore, LINC02159 expression was upregulated in CRC cell lines (Fig. 1B) and 18 paired clinical CRC samples (Fig. 1C). Interestingly, an examination of the GTEx database revealed that despite its upregulation in cancer tissues, higher LINC02159 expression was significantly associated with better overall survival (Fig. 1D) and disease-free survival (Fig. 1E) in CRC patients. This paradoxical pattern was further supported by increased LINC02159 expression in CRC tissues compared to adjacent non-tumour tissues (Fig. 1F) and a notable increase in early-stage CRC samples (Fig. 1G). Collectively, these findings reveal a complex role of LINC02159 in CRC: while it is upregulated in CRC tissues, higher expression levels correlate with better patient outcomes, suggesting that LINC02159 may serve as a compensatory tumour suppressor specifically in KRAS^G12D^ mutated CRC.

### LINC02159 acts as a tumour suppressor in CRC progression

**Fig. 2:**
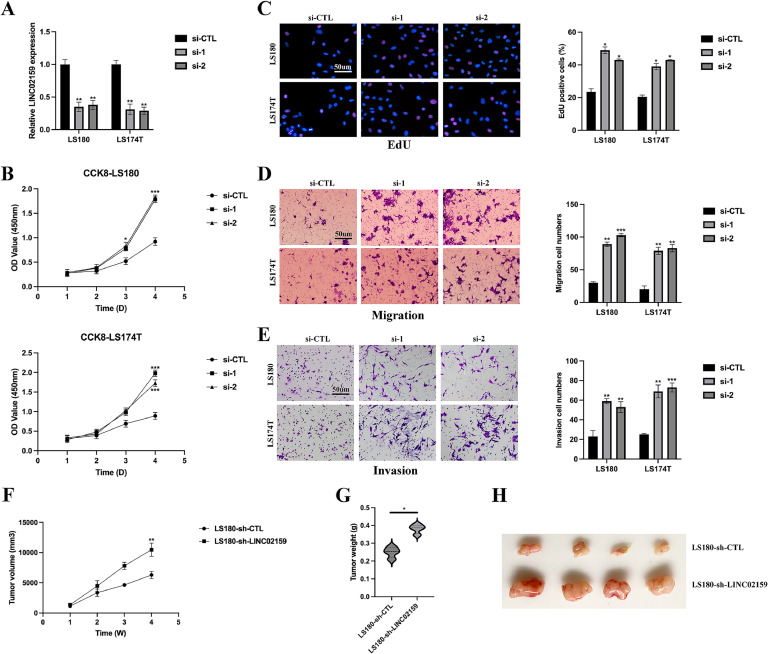
**LINC02159 acts as a tumour suppressor in CRC progression.** A: LINC02159 knockdown cell models were generated by introducing LINC02159 siRNAs into LS180 and LS174T cells. B: Cell proliferation was detected by the CCK-8 assay. C: The EdU assay was utilised to measure cell proliferation ability. Scale bar; 50 μm. D: Cell migration was assessed by a transwell migration experiment. Scale bar; 50 μm. E: Transwell invasion assay was conducted to evaluate cell invasion. Scale bar; 50 μm. The nude mouse xenograft model showed that LINC02159 knockdown increased tumour growth (F) and tumour weight (G) compared to LS180-si-CTL cells. H: Representative images of tumours in nude mice. *P<0.05, **P<0.01, ***P<0.001. Data are presented as mean ± SD.

LINC02159 knockdown cell models were developed to elucidate the role of LINC02159 in CRC progression (Fig. 2A). Subsequent assays employing CCK-8 (Fig. 2B) and EdU incorporation (Fig.2C) demonstrated that LINC02159 suppression significantly enhanced cellular proliferation. Additionally, LINC02159 knockdown facilitated cellular migration (Fig. 2D) and invasion (Figs. 2E). LINC02159 knockdown also increased tumour growth in vivo (Fig. 2F-H). Collectively, these findings indicate that LINC02159 serves as a tumour suppressor in CRC progression.

### MRTX1133 inhibits tumourigenic properties and activates the ferroptotic activity of KRAS^G12D^ mutated CRC by upregulating LINC02159

**Fig. 3:**
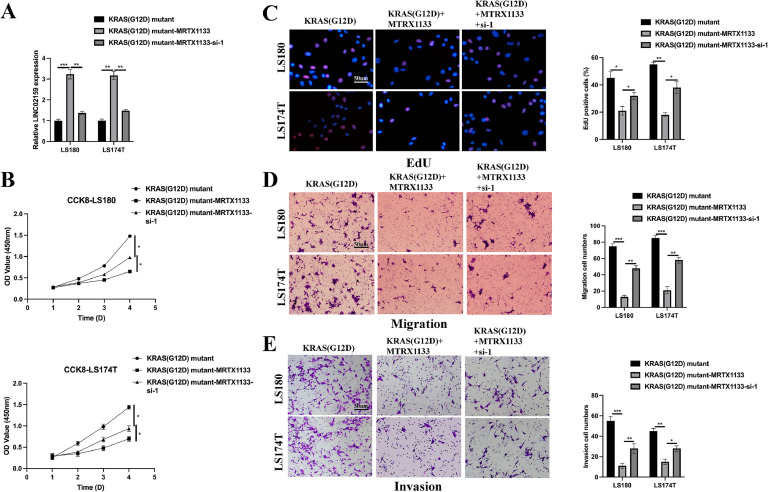
MRTX1133 inhibits the tumourigenic properties of KRAS^G12D^ mutated CRC by upregulating LINC02159.

**Fig. 4:**
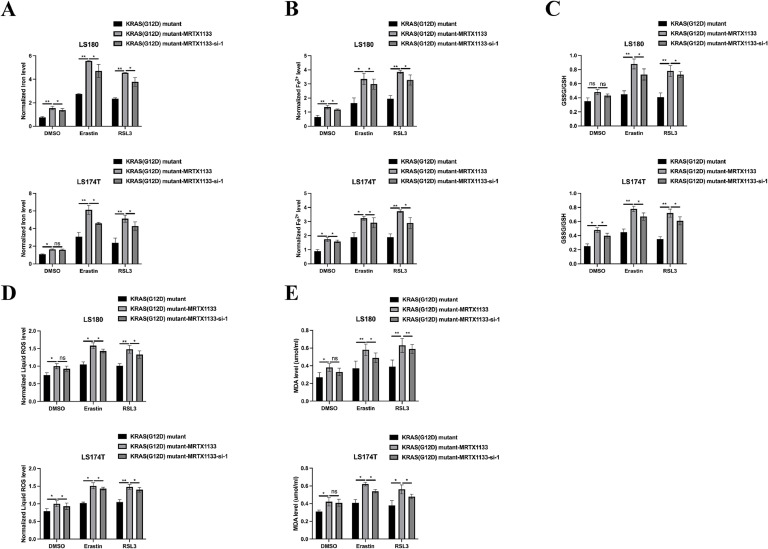
**MRTX1133 activates the ferroptotic activity of KRAS^G12D^ mutated CRC through upregulating LINC02159.** Total iron (A), ferrous iron (B), the ratio of GSSG/GSH (C), lipid ROS (D), and MDA (E) levels in the indicated cell models after Erastin (10 μM) or RSL3 (2 μM) treatment for 12 h ns: no significance, *P<0.05, **P<0.01, ***P<0.001. Data are presented as mean ± SD.

Given the capacity of MRTX1133 to reverse the suppressive effects of the KRAS^G12D^ mutation on LINC02159 expression in CRC cells, we further explored the functional implications of LINC02159 in KRAS^G12D^-mutated CRC cells treated with MRTX1133 (Fig. 3A). The CCK-8 and EdU assay results revealed that LINC02159 knockdown negated the growth-inhibitory effects of MRTX1133 on KRAS^G12D^-mutated CRC cells (Figs. 3B-C). Similarly, MRTX1133 reduced migration and invasion activities induced by the KRAS^G12D^ mutation. However, this reduction was reversed upon LINC02159 knockdown, suggesting that LINC02159 modulates the efficacy of MRTX1133 in these contexts (Figs. 3D-E). Furthermore, considering the established link between KRAS^G12D^ and ferroptosis, we explored whether LINC02159 influences KRAS^G12D^-mediated ferroptosis in the presence of MRTX1133^18–20^. MRTX1133 elevated levels of iron (Fig. 4A), ferrous iron (Fe^2+^) (Fig. 4B), the GSSG to GSH ratio (Fig. 4C), lipid ROS (Fig. 4D), and MDA (Fig. 4E) in KRAS^G12D^-mutated cells. Notably, LINC02159 knockdown attenuated the pro-ferroptotic effects of MRTX1133. These findings highlight the critical role of LINC02159 in mediating the therapeutic effects of MRTX1133 on the malignant properties of KRAS^G12D^-mutated CRC cells.

A: LINC02159 knockdown cell models were generated by introducing LINC02159 siRNAs into KRAS^G12D^ mutated LS180 and LS174T cells upon MRTX1133 treatment. B: Cell proliferation was detected by the CCK-8 assay. C: The EdU assay was utilised to measure cell proliferation ability. Scale bar; 50 μm. D: A transwell migration experiment was performed to measure cell migration. Scale bar; 50 μm. E: A transwell invasion assay was conducted to evaluate cell invasion. Scale bar; 50 μm. *P<0.05, **P<0.01, ***P<0.001. Data are presented as mean ± SD.

### LINC02159 expression is modified by METTL14

**Fig. 5:**
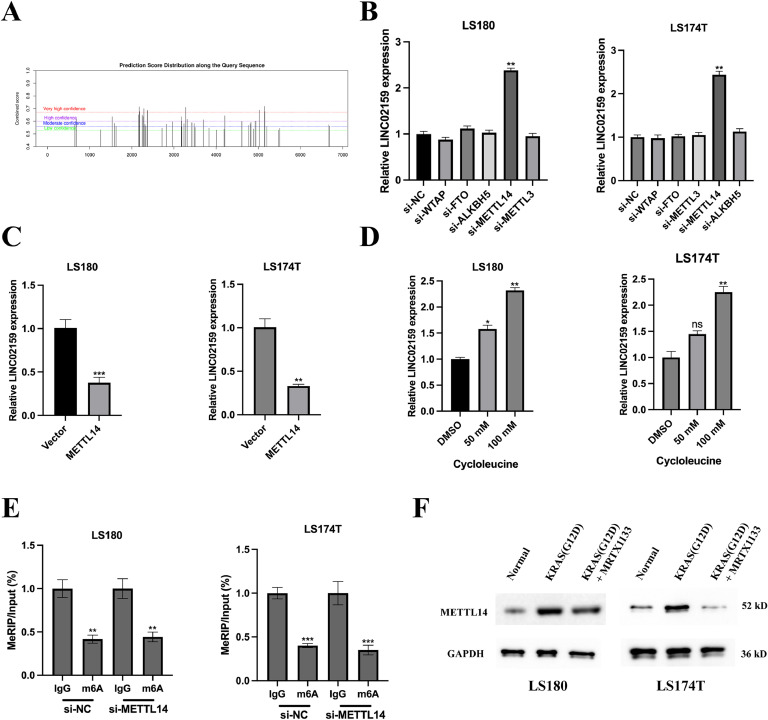
**LINC02159 expression is modified by METTL14.** A: m6A modification sites of LINC02159 were obtained from the JASPAR database. B: Cell models of siRNAs were generated and validated by qRT-PCR. C: LINC02159 expression upon METTL14 overexpression was measured by qRT-PCR in LS180 and LS174T. D: qRT‐PCR to examine LINC02159 expression in cycloleucine‐treated LS180 and LS174T cells. E: MeRIP‐qPCR assay to analyse m6A modification of LINC02159 in LS180 and LS174T after METTL14 knockdown. F: Protein expression of METTL14 in LS180/LS180-KRAS^G12D^ and LS174T/LS174T-KRAS^G12D^ cell upon MRTX1133 or METTL4 siRNA treatments were measured by western blotting. *P<0.05, **P<0.01, ***P<0.001. Data are presented as mean ± SD.

Recently, the prevalent RNA epigenetic regulation of N6-methyladenosine (m6A) on lncRNAs has been studied in-depth[[21], [22], [23], [24]]. Leveraging the SRAMP prediction tool (http://www.cuilab.cn/sramp), our study identified multiple m6A modification sites on the LINC02159 transcript indicative of post-transcriptional regulation (Fig. 5A). To explore the dynamics of m6A modification, we systematically silenced several common m6A modifiers, METTL3, METTL14, WTAP, FTO, and ALKBH5. METTL14 knockdown increased LINC02159 expression (Fig. 5B), while METTL14 overexpression decreased LINC02159 levels (Fig. 5C), suggesting a regulatory role of METTL14 in the m6A modification of LINC02159. Further exploration involved the application of cycloleucine, a known m6A synthesis inhibitor, which induced a dose-dependent increase in LINC02159 RNA levels (Fig. 5D), confirming the involvement of m6A modifications in regulating LINC02159 stability or degradation. Methylated RNA immunoprecipitation (MeRIP) PCR analysis showed that METTL14 silencing not only reduced m6A on LINC02159 but also enhanced its expression (Fig. 5E), suggesting that METTL14 directly affects the m6A landscape of LINC02159, influencing its cellular levels. Moreover, MRTX1133 significantly reduced METTL14 expression in cells pronounced by the KRAS^G12D^ mutation (Fig. 5F). These findings delineate a clear role of METTL14 in modulating the m6A modification of LINC02159, which in turn impacts its expression in CRC cells.

### METTL14 is crucial for the role of MRTX1133 in KRAS^G12D^ mutated CRC

**Fig. 6:**
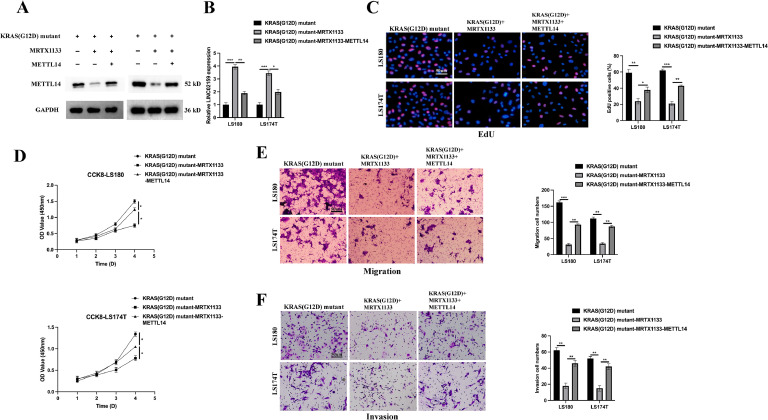
**METTL14 is crucial for the tumourigenic inhibitive role of MRTX1133 in KRAS^G12D^ mutated CRC.** A-B: METTL14 overexpression cell models were generated by introducing METTL14 vectors into KRAS^G12D^ mutated LS180 and LS174T cells upon MRTX1133 treatment, METTL14 and LINC02159 expression were validated by western blot and qRT-PCR. C: EdU assay was utilised to measure cell proliferation ability. Scale bar; 50 μm. D: Cell proliferation was detected by CCK-8 assay. Scale bar; 50 μm. E: A transwell migration experiment was performed to measure cell migration. Scale bar; 50 μm. F: A transwell invasion assay was conducted to evaluate cell invasion. *P<0.05, **P<0.01, ***P<0.001. Data are presented as mean ± SD.

**Fig. 7:**
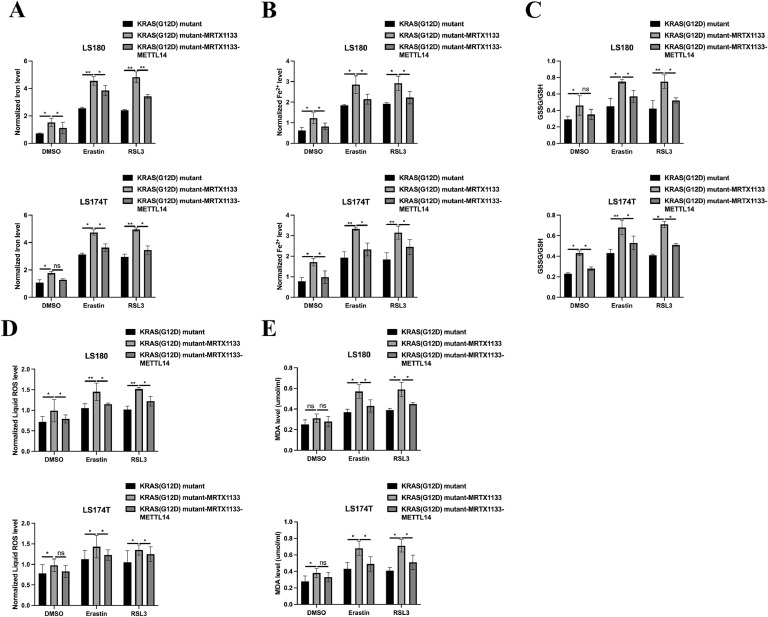
**METTL14 is vital for the pro-ferroptotic role of MRTX1133 in KRAS^G12D^ mutated CRC.** Total iron (A), ferrous iron (B), the ratio of GSSG/GSH (C), lipid ROS (D), and MDA (E) in the indicated cell models after Erastin (10 μM) or RSL3 (2 μM) treatment for 12 h ns: no significance, *P<0.05, **P<0.01, ***P<0.001. Data are presented as mean ± SD.

Specialised cell models were developed to investigate the role of METTL14 in modulating the response of KRAS^G12D^-mutated CRC cells to MRTX1133 (Fig. 6A-B). CCK-8 and EdU assays revealed that METTL14 counteracts the growth-inhibitory effects of MRTX1133 on KRAS^G12D^-mutated CRC cells (Fig. 6C-D), suggesting a complex interaction where METTL14 potentially stabilises or modifies expression that contributes to cellular proliferation despite the presence of MRTX1133. Transwell assays demonstrated that while MRTX1133 effectively reduced migration and invasion activities associated with the KRAS^G12D^ mutation, these reductions were significantly reversed upon METTL14 overexpression (Fig. 6E-F). This indicates that METTL14 modifies the therapeutic efficacy of MRTX1133, potentially by altering the cellular processes. Also, METTL14 overexpression mitigated the pro-ferroptotic effects induced by MRTX1133 (Fig. 7A-E), further supporting its pivotal role in influencing the cellular response to the drug. Together, these findings underscore the critical influence of METTL14 in shaping the therapeutic response of KRAS^G12D^-mutated CRC cells to MRTX1133.

### LINC02159 stabilises FOXC2 expression via de-ubiquitination

**Fig. 8:**
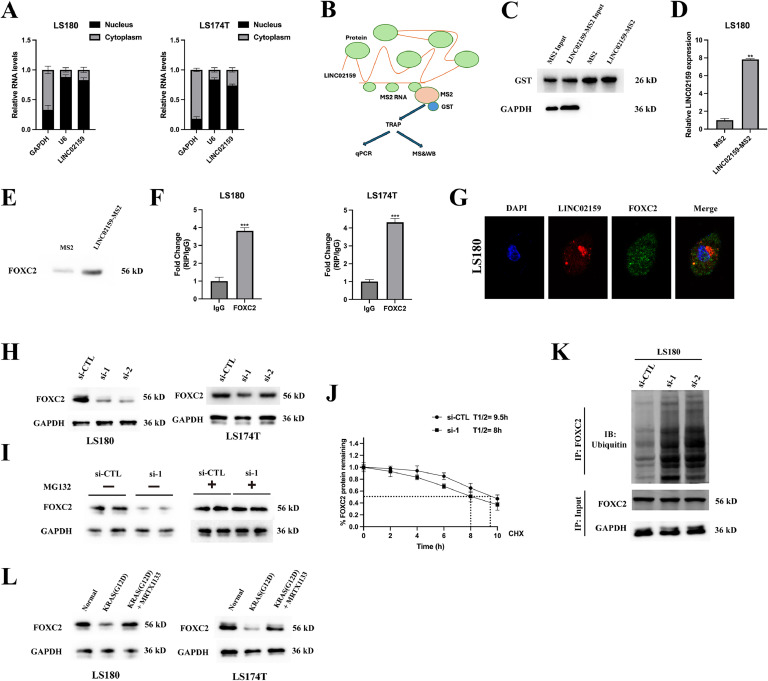
**LINC02159 stabilises FOXC2 expression via de-ubiquitination.** A: Cellular distribution of LINC02159 in LS180 and LS174T cells. B: Schematic diagram of the TRAP experiment. C: Western blotting to detect GST expression after TRAP. D qRT-PCR to measure LINC02159 expression in the product after TRAP. E: LINC02159 binding to FOXC2 was confirmed by western blotting. F: RIP experiments for the interaction between FOXC2 and LINC02159. G: FISH and immunofluorescence staining for the co-localisation of LINC02159 and FOXC2 in LS180 cells. H: Western blotting to measure FOXC2 expression in LINC02159 downregulated LS180 and LS174T cells. I: A western blot assay was utilised to measure FOXC2 expression in LINC02159 downregulated LS180 and LS174T cells with/without 8-hour MG132 (20 μM) treatment. J: The intensity of endogenous FOXC2 expression in cells treated with 50 mg/ml CHX for each time point was quantified by densitometry. K: upon MG132 treatment, FOXC2 was immunoprecipitated with an anti-FOXC2 antibody and the ubiquitination of FOXC2 was examined by western blotting. L: FOXC2 expression in LS180/LS180-KRAS^G12D^ and LS174T/LS174T-KRAS^G12D^ cells upon MRTX1133 treatments were measured by western blotting. **P<0.01, ***P<0.001. Data are presented as mean ± SD.

To further elucidate the molecular mechanisms involving LINC02159, we investigated its downstream effectors. Initial studies determined that LINC02159 predominantly resides in the nucleus of CRC cells (Fig. 8A) and the TRAP assay identified proteins interacting with LINC02159 confirming its nuclear localisation (Figs. 8B-D). Mass spectrometry highlighted several proteins enriched in the LINC02159-MS2 bait group compared to the control MS2 group, with FOXC2 showing the highest fold enrichment. Based on previous studies highlighting the importance of FOXC2 in cancer progression[25,26], we investigated its potential interaction with LINC02159. Subsequent verification through an independent TRAP assay confirmed the interaction between LINC02159 and FOXC2 (Fig. 8E). RIP assays further validated that LINC02159 was enriched in the FOXC2-immunoprecipitated complex in CRC cells (Fig. 8F). Immunofluorescent staining demonstrated co-localisation of LINC02159 with FOXC2 in LS180 cells (Fig. 8G). Functional assays showed that knockdown of LINC02159 led to a reduction in FOXC2 protein levels in CRC cells (Fig. 8H). Intriguingly, treatment with the proteasome inhibitor MG-132 restored FOXC2 expression in LINC02159 knockdown cells to levels observed in control cells (Fig. 8I), indicating that LINC02159 may regulate FOXC2 expression via a ubiquitination-dependent pathway. Further investigation of the FOXC2 stability revealed that its half-life was reduced from 9.5 hours in control cells to 8 hours in LINC02159 knockdown cells (Fig. 8J). Analysis of ubiquitination levels demonstrated increased ubiquitination of the endogenous protein FOXC2 following LINC02159 knockdown (Fig. 8K). Moreover, there was a decrease in FOXC2 expression in KRAS^G12D^-mutated CRC cells, which was reversed by MRTX1133 treatment suggesting that the KRAS^G12D^ mutation may negatively impact FOXC2 expression and that MRTX1133 could mitigate this effect (Fig. 8L). These findings suggest that LINC02159 plays a significant role in regulating the stability and expression of FOXC2 through a ubiquitination-mediated pathway.

### Identifying METTL14/LINC02159/FOXC2 signal upon MRTX1133 treatment in KRAS^G12D^ mutated CRC

**Fig. 9:**
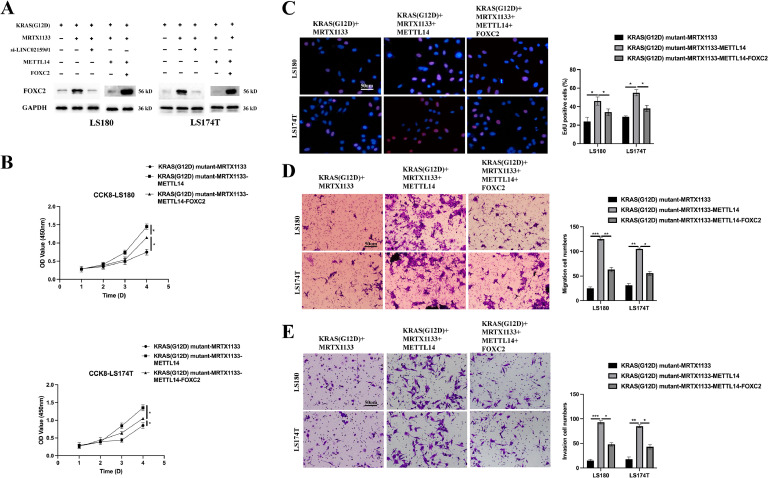
**The METTL14/LINC02159/FOXC2 signal upon MTRX1133 treatment in the tumourigenic properties of KRAS^G12D^ mutated CRC.** A: Western blotting to detect FOXC2 expression in the indicated cells. B: Cell proliferation was detected by the CCK-8 assay. C: The EdU assay was utilised to measure cell proliferation. Scale bar; 50 μm. D: A transwell migration experiment was performed to measure cell migration. Scale bar; 50 μm. E: A transwell invasion assay was conducted to evaluate cell invasion. Scale bar; 50 μm. *P<0.05, **P<0.01, ***P<0.001. Data are presented as mean ± SD.

**Fig. 10:**
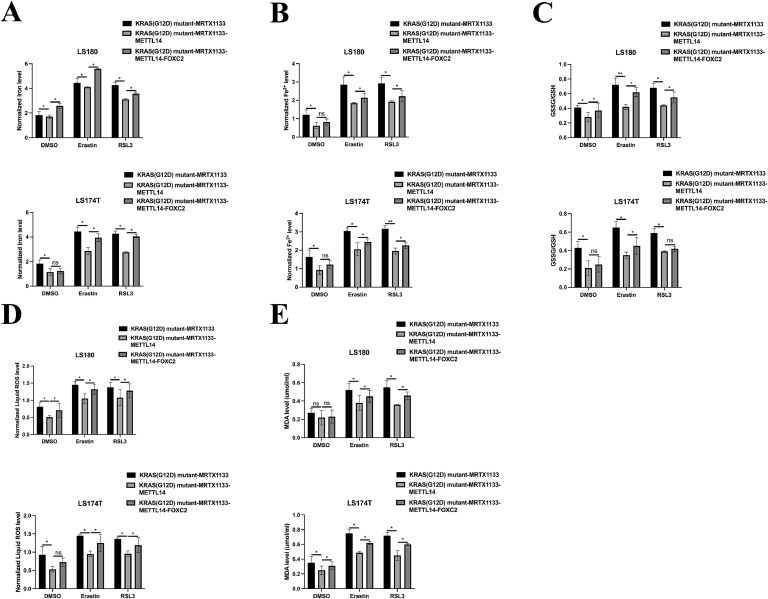
**The METTL14/LINC02159/FOXC2 signal upon MTRX1133 treatment in the ferroptosis activity of KRAS^G12D^ mutated CRC.** Total iron (A), ferrous iron (B), the ratio of GSSG/GSH (C), lipid ROS (D), and MDA (E) in the indicated cell models after Erastin (10 μM) or RSL3 (2 μM) treatment for12 h. ns: no significance, *P<0.05, **P<0.01, ***P<0.001. Data are presented as mean ± SD.

To elucidate the complex signalling interactions between MRTX1133, METTL14, LINC02159, and FOXC2 in modulating the response of KRAS^G12D^-mutated CRC cells, we established and validated specific cell models (Fig. 9A). CCK-8 and EdU assays revealed that FOXC2 counteracts the growth-promotive effects that METTL14 exerts in the presence of MRTX1133 on KRAS^G12D^-mutated CRC cells (Figs. 9B-C). Transwell assays showed that while METTL14 significantly induced migration and invasion in KRAS^G12D^-mutated cells treated with MRTX1133, these effects were substantially reversed upon FOXC2 overexpression (Figs. 9D-E). Notably, FOXC2 overexpression mitigated the ferroptotic effects induced by METTL14 (Fig. 10A-E), suggesting that FOXC2 might be a key regulator in shielding cells from ferroptotic cell death triggered by METTL14, further complicating the cellular dynamics in KRAS^G12D^-mutated CRC cells treated with MRTX1133.

## Discussion

KRAS is the predominant isoform among RAS mutations[27], occurring frequently in various cancers, particularly CRC. Developing strategies to target KRAS mutant proteins has proved challenging due to the lack of a deep binding pocket on the KRAS mutant protein[28,29]. MRTX1133 has recently emerged as a high-affinity, mutation-selective, non-covalent KRAS^G12D^ inhibitor[30,31]. The feedback activation of epidermal growth factor receptor (EGFR)-mediated wild-type RAS signalling contributes to the reduced effectiveness of KRAS^G12D^ inhibitor therapy in CRC, unveiling the role of MRTX1133 in KARS^G12D^ mutant CRC[17]. Moreover, given the vital role of lncRNAs in CRC progression, we hypothesised that MRTX1133 might affect KRAS^G12D^ mutant CRC progression by regulating lncRNAs. Analysis of a GEO dataset revealed that LINC02159 was highly upregulated upon MRTX1133 treatment.

LINC02159 functions as an oncogene by promoting NSCLC tumourigenesis progression both in vivo and *in vitro*[*32*]. Interestingly, our study revealed that LINC02159 exhibits a context-dependent role in CRC, where it functions as a tumour suppressor specifically in KRAS^G12D^-mutant CRC treated with MRTX1133. The METTL14/LINC02159/FOXC2 axis appears to be specifically activated in this context, as demonstrated by MRTX1133′s ability to modulate METTL14 expression, enhance LINC02159 levels, and stabilize FOXC2 protein through deubiquitination. Notably, LINC02159 expression is downregulated in KRAS^G12D^-mutant CRC cells, thereby compromising its tumour-suppressive function. Accumulating studies suggest that ferroptosis exerts a crucial role in KRAS^G12D^-mutant CRC [[18], [19], [20]], thus we explored whether MRTX1133 and LINC02159 are associated with ferroptotic activity in KRAS^G12D^-mutant CRC. Our findings suggested that MRTX1133 promoted ferroptosis in KRAS^G12D^-mutant CRC cells, whilst this effect could be alleviated by LINC02159 knockdown. These results highlight a novel direction for investigating the biological role of MRTX1133 during the inhibition of KRAS^G12D^-mutant CRC.

Considering the significant role of m6A modification in lncRNAs expression [21,22], we investigated the upstream regulator of LINC02159, demonstrating that METTL14 negatively regulates LINC02159 expression in CRC cells by modifying the m6A level of LINC02159. It has been reported that METTL14 acts as a tumour suppressor gene in CRC progression [33,34]. However, the present study revealed that METTL14 expression was upregulated in KRAS^G12D^-mutant CRC cells and decreased by MRTX1133. METTL14 promoted KRAS^G12D^-mutant CRC tumourigenesis and inhibited ferroptosis activities, phenomena which could be reversed by MRTX1133 treatment. Moreover, the expression of FOXC2, a protein downstream of LINC02159, was stabilised by LINC02159 via direct interaction and de-ubiquitination. The rescue experiments confirmed the novel METTL14/LINC02159/FOXC2 signal in KRAS^G12D^-mutant CRC progression after MRTX1133 treatment.

Limitations: due to the limited availability of clinical samples, we were unable to analyse the clinical significance of and the correlation between METTL14, LINC02159, and FOXC2. Another limitation is the unsuccessful construction of KRAS^G12D^-mutant CRC animal models which prevented in vivo validation of METTL14, LINC02159, and FOXC2. While our study demonstrates the METTL14/LINC02159/FOXC2 axis is crucial for MRTX1133′s effects in KRAS^G12D^-mutated CRC, whether this pathway directly affects KRAS^G12D^ protein levels remain to be investigated.

In summary, we identified a METTL14/LINC02159/FOXC2 signalling axis that is specifically activated in response to MRTX1133 treatment in KRAS^G12D^-mutated CRC cells. Our findings demonstrate that this pathway functions as an integrated signalling unit, where MRTX1133 treatment leads to decreased METTL14 expression, resulting in enhanced LINC02159 stability and subsequent FOXC2 protein stabilization through deubiquitination. This context-specific activation of the METTL14/LINC02159/FOXC2 axis represents a promising therapeutic target for developing more effective treatment strategies for KRAS^G12D^-mutated CRC.

## Funding

This work was funded and supported by the Key Project in Natural Science Research in Higher Education Institutions of Anhui Province (2023AH051777) , the Key project of Natural Science Foundation of Wannan Medical College (WK2022ZF29), the Key research project of Wuhu Municipal Health Commission (WHWJ2023z007) and the Climbing Scientific Peak Project for Talents, the Second Affiliated Hospital of Wannan Medical College (DFJH2022018).

## Ethics approval and consent to participate

The study was conducted in accordance with the Declaration of Helsinki (as revised in 2013). The study was approved by ethics board of The Second Affiliated Hospital of 10.13039/501100014980Wannan Medical College (WYEFYLS2024137) and individual consent for this retrospective analysis was waived.

## Informed consent statement

Informed consent was obtained from all subjects involved in the study. Written informed consent has been obtained from the patient(s) to publish this paper.

## Consent for publication

Not applicable.

## Data availability

Study data can be obtained from the corresponding author upon reasonable request.

## CRediT authorship contribution statement

**Junwei Zou:** Writing – original draft. **Xiuhua Shi:** Validation. **Zhaoying Wu:** Formal analysis. **Siyuan Zuo:** Investigation. **Xiaolei Tang:** Methodology. **Hailang Zhou:** Conceptualization. **Yong Huang:** Conceptualization.

## Declaration of competing interest

The authors declare that they have no known competing financial interests or personal relationships that could have appeared to influence the work reported in this paper. The author is an Editorial Board Member/Editor-in-Chief/Associate Editor/Guest Editor for *[Journal name]* and was not involved in the editorial review or the decision to publish this article.

## Appendix Supplementary materials

Image, application 1
