# Supplementary figures and images for: MRTX1133 attenuates KRASG12D mutated-colorectal cancer progression through activating ferroptosis activity via METTL14/LINC02159/FOXC2 axis
Source: Transl Oncol. 2024 Dec 9;52:102235. doi: 10.1016/j.tranon.2024.102235 (PMC11683245; doi:10.1016/j.tranon.2024.102235)

**Figure 5**

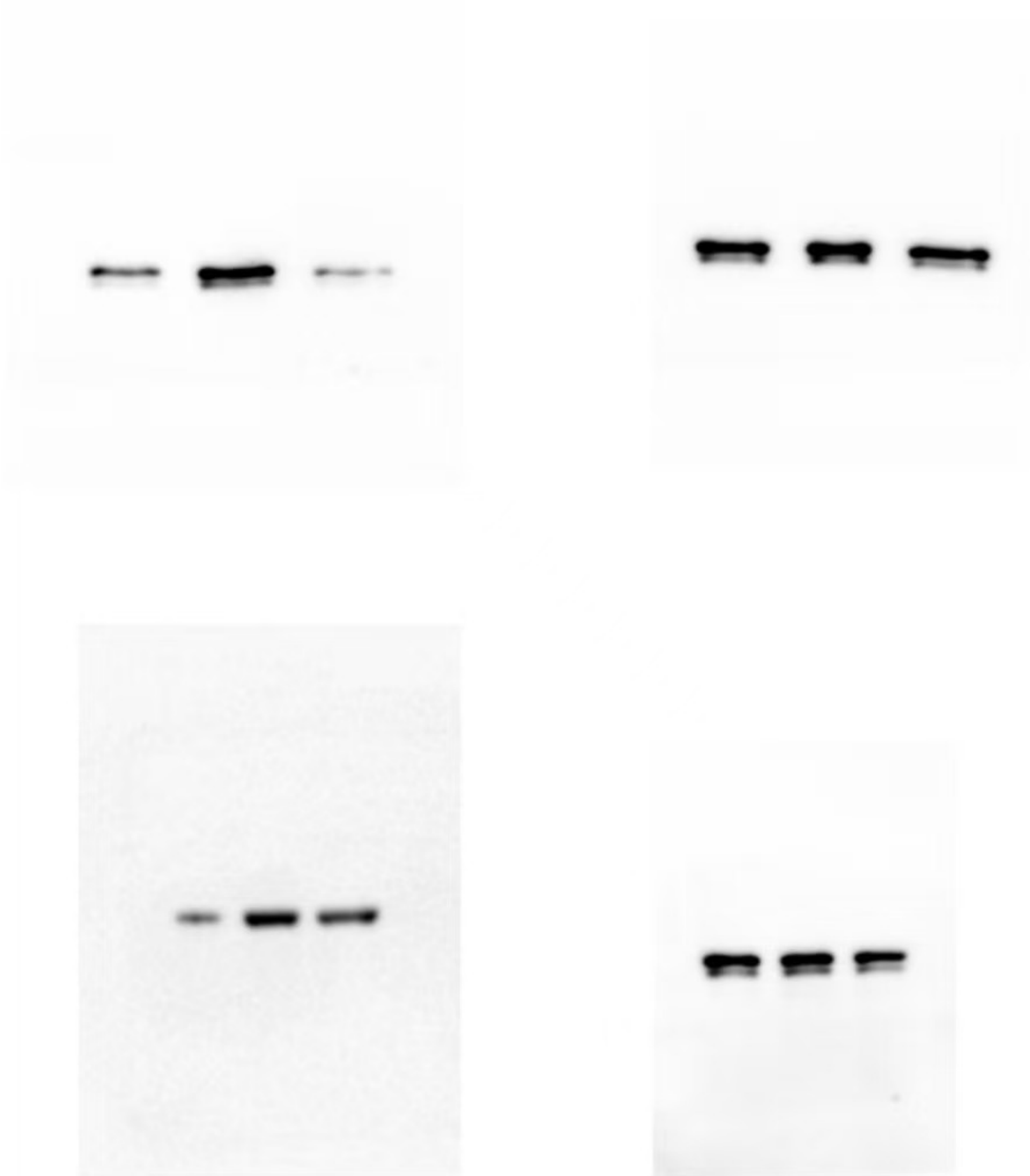

**Figure 8**

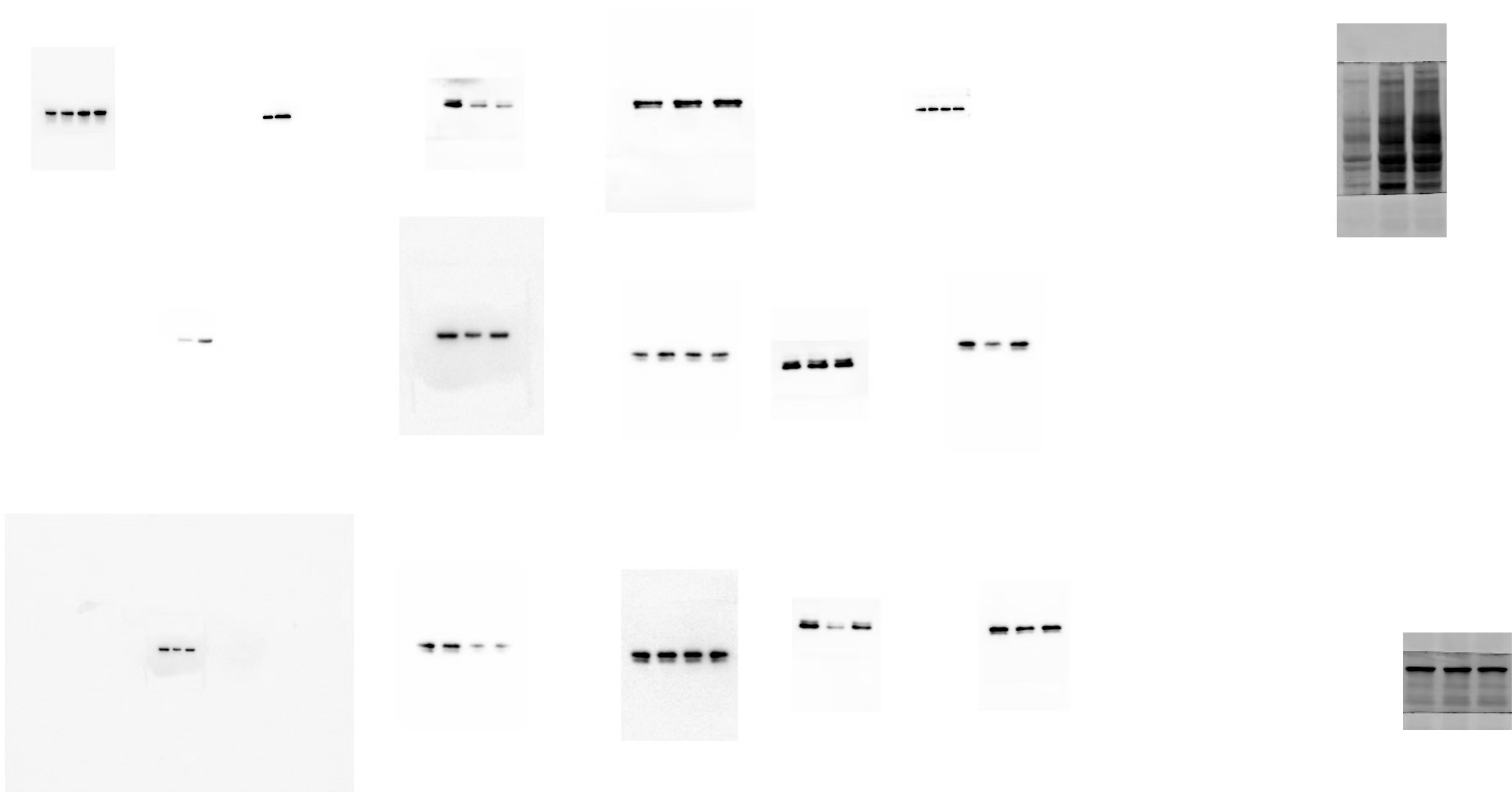

**Figure 6**

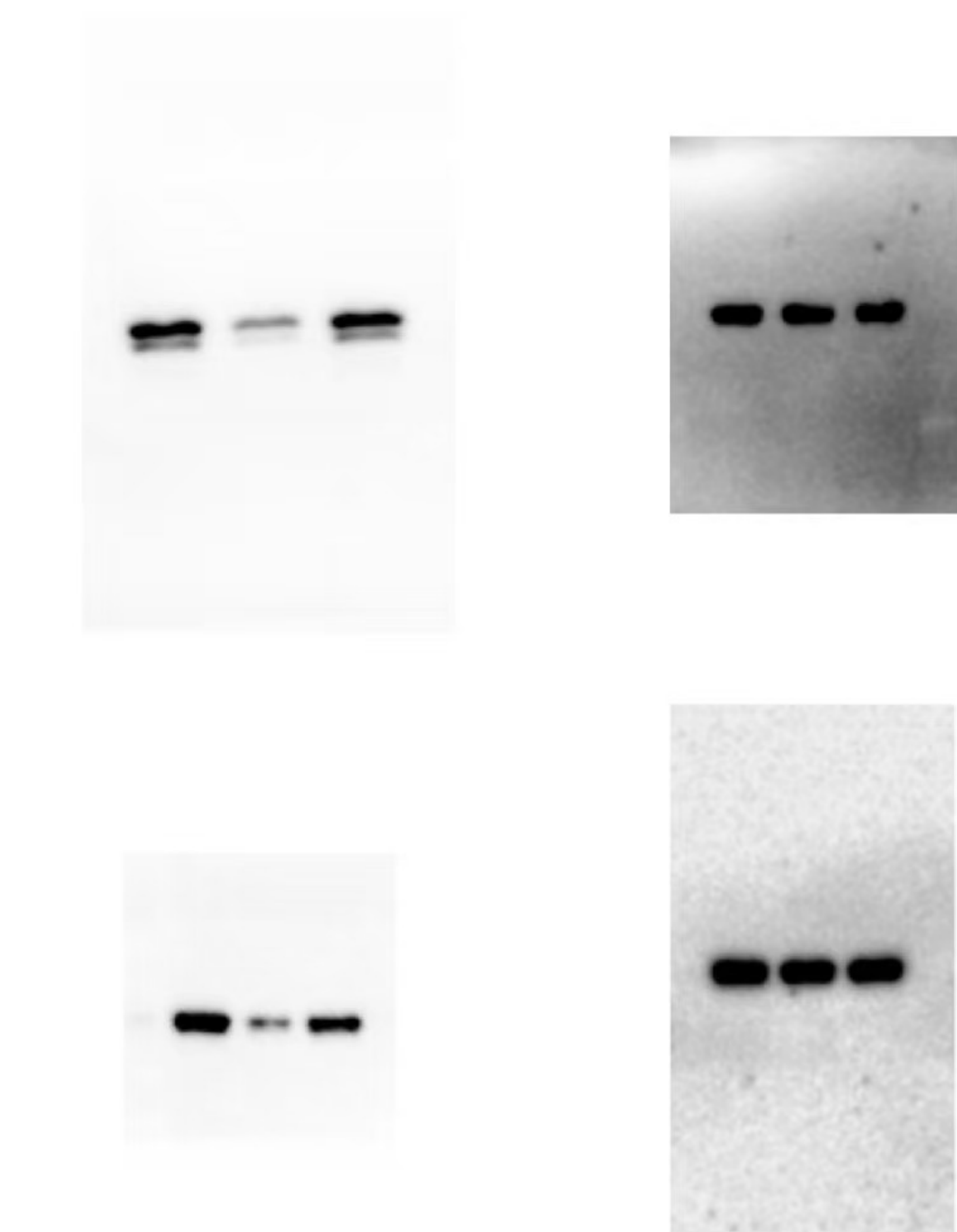

**Figure 9**

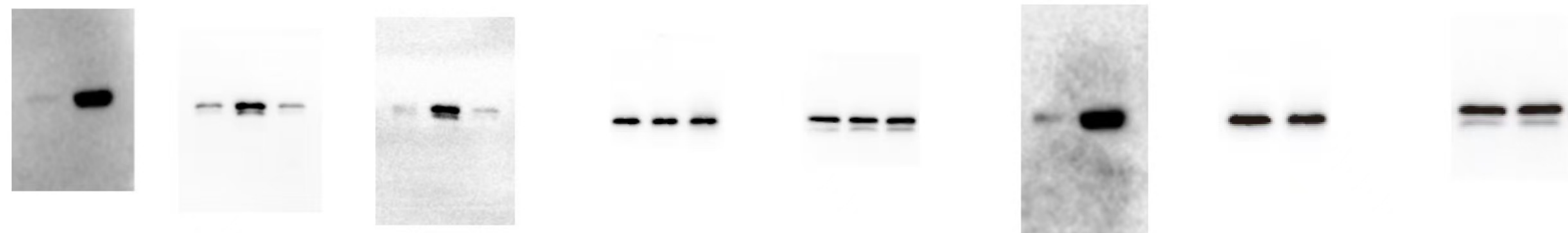

Supplement: Supplementary file 1 [file mmc1.pdf]
